# The value of CSF flow studies in the management of CSF disorders in children: a pictorial review

**DOI:** 10.1186/s13244-019-0686-x

**Published:** 2019-01-28

**Authors:** Shaimaa Abdelsattar Mohammad, Noha Mohamed Osman, Khaled A. Ahmed

**Affiliations:** Department of Radiodiagnosis, Pediatric Radiology section, Faculty of Medicine, Ain-Shams University, Abbasia, Cairo, 11657 Egypt

**Keywords:** Pediatric hydrocephalus, Ventricular dilatation ex-vacuo, Phase contrast, Blake’s pouch and arachnoid cyst, Dandy-Walker malformation

## Abstract

CSF flow disorders are frequently encountered in children. The advent of MR technology with the emergence of new pulse sequences allowed better understanding of CSF flow dynamics. In this pictorial review, we aim to conduct a comprehensive review of the MR protocol used to study CSF flow disorders and to discuss the utility of each pulse sequence in the adopted protocol. We will focus on the key anatomical structures that should be examined to differentiate hydrocephalus form ventricular dilatation ex-vacuo. The MR features of obstructive and communicating hydrocephalus will be discussed, in addition to the manifestations of CSF disorders associated with posterior fossa malformations (Dandy-Walker malformation, Chiari, and Blake’s pouch cyst). Moreover, the value of MRI in the assessment of patients following interventional procedures (ventriculoperitoneal shunt and third ventriculostomy) will be addressed.

**Electronic supplementary material:**

The online version of this article (10.1186/s13244-019-0686-x) contains supplementary material, which is available to authorized users.

## Key points

- MRI is the ideal modality to investigate CSF flow disorders in children.
- Our practical illustrated protocol based on 3D high-resolution T2-WI and phase contrast along with conventional sequences helps to recognize different entities of CSF flow disorders in children.
- Most cases of hydrocephalus are related to obstruction with the aqueduct of Sylvius being the commonest site.

## Introduction

The advent in MRI technology has led to better understanding of CSF flow disorders which are frequently encountered in children [1, 2]. Flow-sensitive MRI techniques and three-dimensional high-resolution sequences have been applied for functional and anatomical assessment of CSF flow dynamics, respectively [3–5]. These studies can discriminate between communicating and non-communicating hydrocephalus with accurate localization of the site of obstruction [6]. Assessment of arachnoid cysts and determination of CSF movement alterations in cases with Chiari and Dandy-Walker malformations can be properly performed [3, 7]. Moreover, hydrocephalus and ventriculomegaly ex-vacuo can be differentiated. In addition to shunt surgeries, interventional procedures such as endoscopic third ventriculostomy and aqueductoplasty are among the treatment options. Accurate diagnosis of the site of obstruction and detection of a combination of pathologies are crucial for proper selection of the treatment strategy and improving the postoperative outcome [7, 8]. In this review, we will describe the MRI protocol adopted for the assessment of the ventricular system and CSF flow in children and the value of each imaging sequence in order to help reach the diagnosis. In addition, imaging features of various disorders are described.

## MRI protocol

**Fig. 1:**
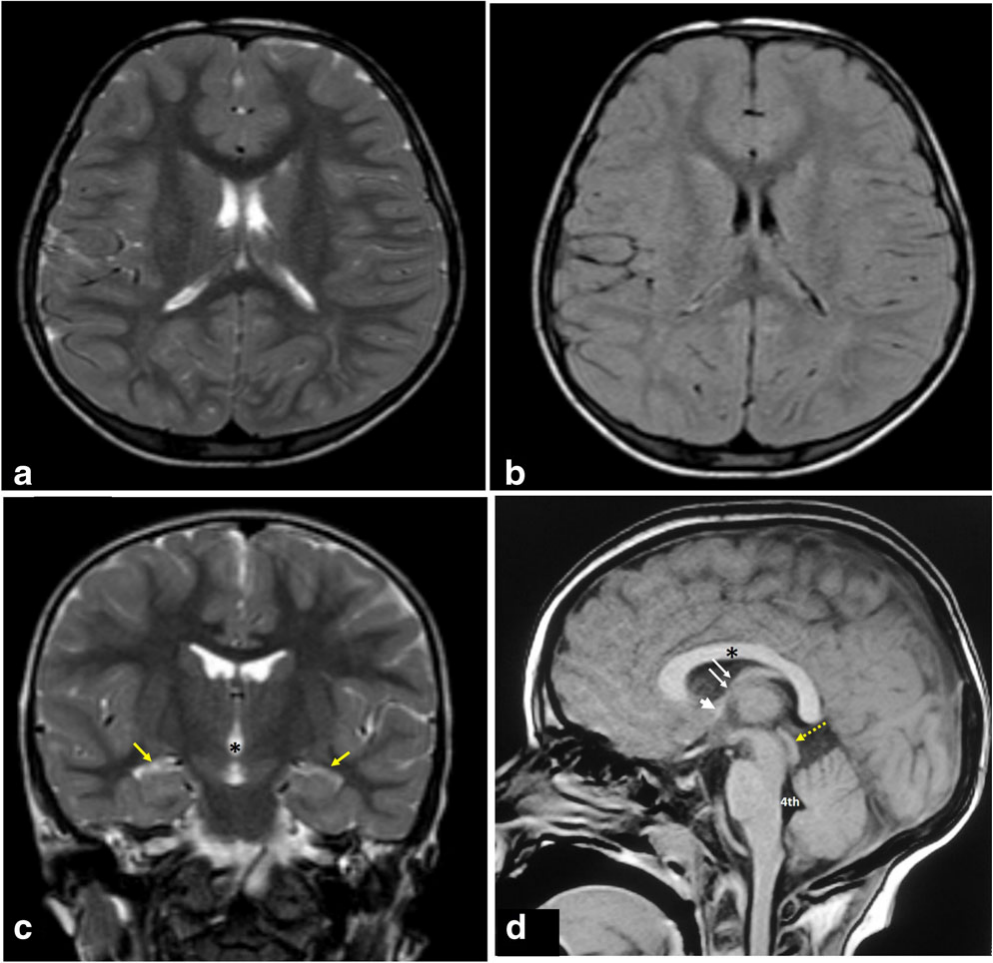
MRI protocol for hydrocephalus. Normal images of a 3-year-old boy: **a** Axial T2-WI. **b** Axial FLAIR. **c** Coronal T2-WI through the lateral and third ventricles (asterisk); note the morphology of the ventricular system, especially the temporal horns (arrows). **d** Midline sagittal T1-WI image shows the corpus callosum (asterisk), the anterior commissure (short arrow), and the fornix (long arrows). The midbrain with aqueduct and tectal plate (dotted arrow), pons, fourth ventricle (4th), and vermis are well seen

**Fig. 2:**
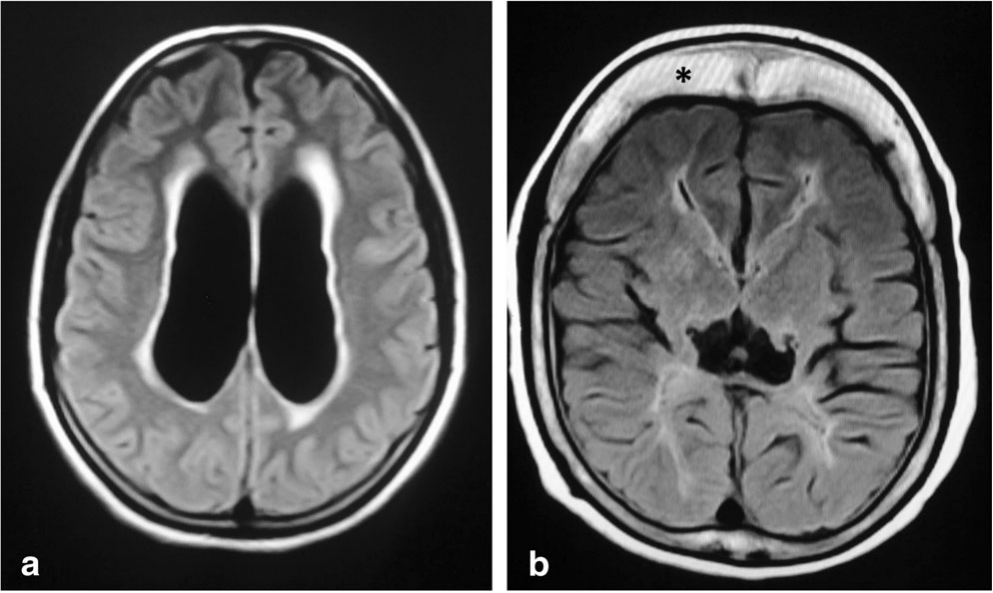
The value of FLAIR sequence in two different patients. **a** Axial FLAIR of a 14-year-old boy showing periventricular high signal in a setting of acute hydrocephalus secondary to a colloid cyst (not shown). **b** Axial FLAIR of an 11-year-old girl with periventricular high signal reflecting poor myelination due to the noxious effect of hydrocephalus. Note the thick skull with prominent diploic space (asterisk) in the chronic shunted patient

**Fig. 3:**
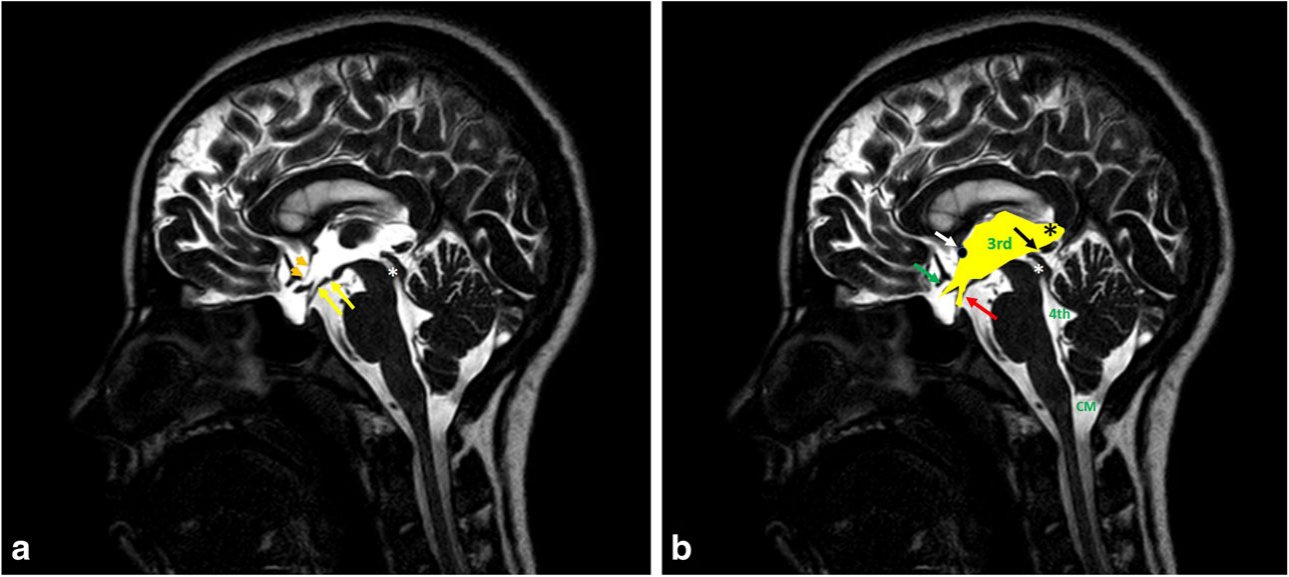
**a**, **b** Sagittal 3D-DRIVE of a 12-year-old boy demonstrates the anatomy of midline structures: the third ventricle (3rd), aqueduct of Sylvius (white asterisk), fourth ventricle (4th), and cisterna magna (CM). Note the concave floor of the third ventricle (yellow arrows) and lamina terminalis (orange arrow heads), chiasmatic and infundibular recesses of the third ventricle (green, red arrows), pineal (black arrow) and suprapineal (asterisk) recesses of the third ventricle, and anterior commissure (white arrow)

The MRI protocol should include brain imaging with T2-weighting in axial and coronal planes, axial fluid attenuation inversion recovery (FLAIR), and sagittal T1-weighted image (T1-WI) (Figs. 1 and 2). High-resolution heavily T2-weighted volumetric sequence (as three-dimensional driven equilibrium 3D-DRIVE) is acquired in sagittal plane. The field of view extends from the left foramen of Luschka to the right one (Fig. 3). Post-contrast T1-WI may be acquired in three planes in cases with intracranial neoplasms or in suspected inflammatory process. Axial T2*-WI may be acquired for better detection of intracranial hemorrhage as a cause of hydrocephalus. Table 1 summarizes our 1.5 T MRI protocol for patients with CSF flow disorder, and Table 2 summarizes the value of each pulse sequence in the recommended protocol.

**Table 1:** MRI protocol

Sequence	3-plane localizer	T2-WI	FLAIR	T1-WI	3D-DRIVE	Phase contrast	Phase contrast	Post-contrast T1-WI	T2*-WI
Slice orientation	Axial, coronal, sagittal	Axial, coronal	Axial	Sagittal	Sagittal**	Sagittal	axial	Axial, coronal and sagittal	Axial
Slice thickness (mm)	10	4	4	4	2	10	4	4	4
Gap (mm)	20	5	5	5	1	10	4	5	5
FOV (cm)	25 × 25	25 × 25	25 × 25	25 × 25	23 × 23	25 × 25	10 × 10	25 × 25	25 × 25
TR (ms)	15	4250	11,000	110	1500	20	18	520	690
TE (ms)	5	100	130	1.8	250	7	11	15	23
Matrix	125 × 256	197 × 256	197 × 256	256 × 256	270 × 340	180 × 256	145 × 200	197 × 256	197 × 256
Flip angle	20	90	90	80	90	10	15	80	18
NEX	1	1	2	1	2	2	2	1	1
Velocity encoding (cm/s)	–	–	–	–	–	20	20	–	–

*3D-DRIVE* three-dimensional driven equilibrium, *FOV* field of view, *NEX* number of excitations

^**^Can be acquired in coronal plane in cases with suspected foramen of Monro stenosis

**Table 2:** Role of MR imaging sequences in the evaluation of the CSF flow abnormalities in children

Sequence	Plane	Key application
T2-WI	Axial	Determination of ventriculomegaly and any other structural abnormalities
FLAIR	Axial	Determination of periventricular abnormal signal which may represent interstitial edema in cases of acute hydrocephalus or secondary to defective myelination in chronic hydrocephalus (Fig. 2)
T2-WI	Coronal	Determination of the size of the temporal horns of both lateral ventricles in relation to the body (Fig. 7)
T2*-WI	Axial	Better detection of intracranial hemorrhage as a cause of hydrocephalus
3D-DRIVE	Sagittal with MPR	Demonstration of the anatomical details of the ventricular system, third ventricular recesses, aqueduct of Sylvius, subarachnoid space, and basal cisterns and to search for adhesions especially in the aqueduct of Sylvius, fourth ventricular exit, and basal subarachnoid space (Figs. 3, 8 and 12)
T1-WI	Sagittal	Determination of anatomy and any structural abnormality
Phase contrast	Sagittal	Qualitative assessment of CSF flow through the aqueduct of Sylvius and basal cisterns (Fig. 4)
Phase contrast	Axial	Quantification of CSF flow and velocity (Fig. 5)
Post-contrast T1-WI^#^	Axial, coronal, sagittal	Detection of abnormal enhancement in cases of suspected inflammatory or neoplastic pathologies (Fig. 9)

*FLAIR* fluid attenuation inversion recovery, *MPR* multiplanar reformat, *3D-DRIVE* three-dimensional driven equilibrium, *T1-WI* T1-weighted image, *T2-WI* T2-weighted image

^#^Optional depending on the indication

**Fig. 4:**
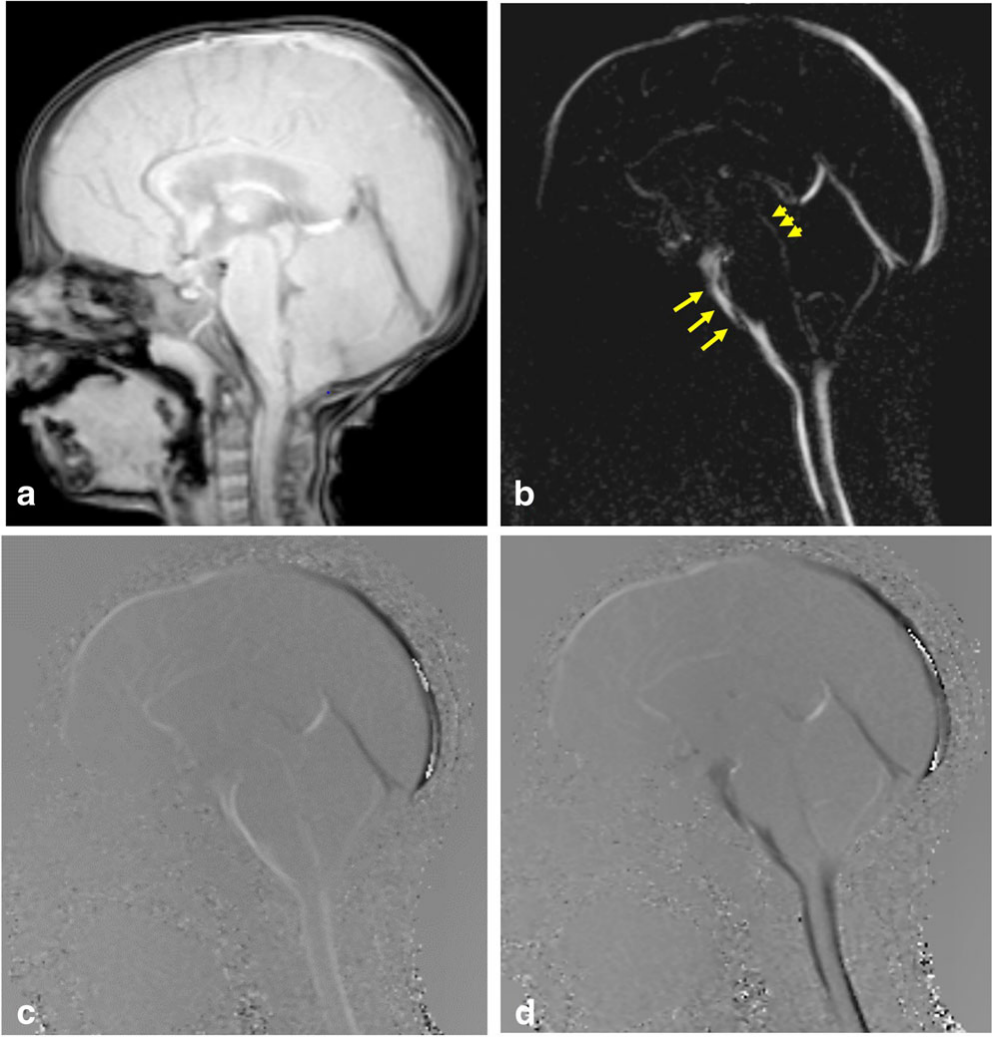
Phase-contrast sequence in sagittal plane of a 2-year-old girl. **a** Re-phased image in which there is visible background. **b** Magnitude image in which the flow is bright with suppressed background. **c**, **d** Phase image demonstrates the to-and-fro (pulsatile) bright and black flow of CSF throughout the cardiac cycle. Note the CSF flow in the prepontine-premedullary cisterns (long arrows) and the aqueduct of Sylvius (short arrows)

**Fig. 5:**
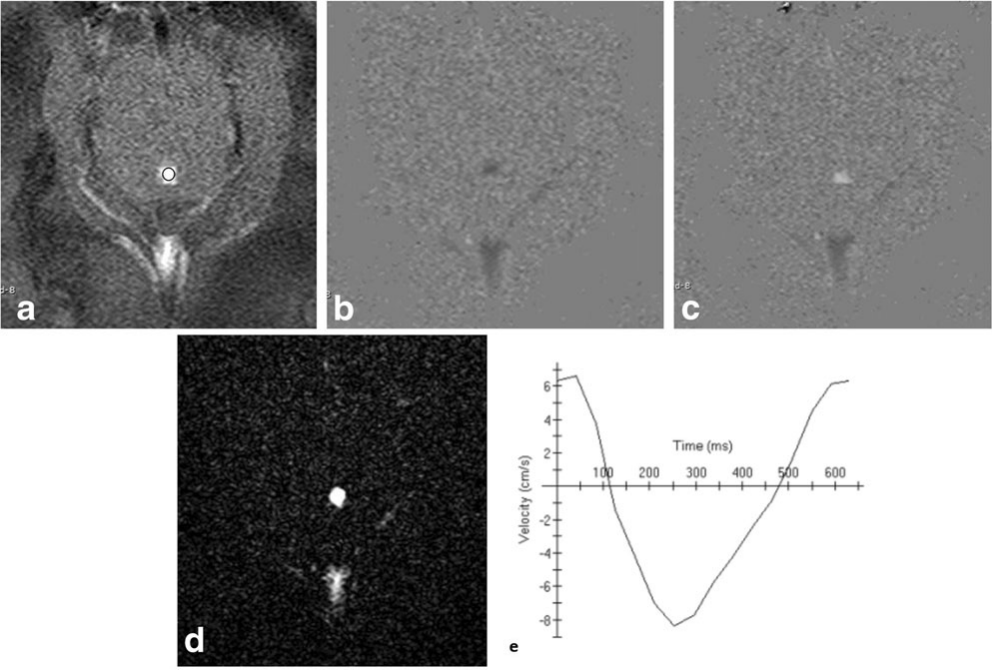
Phase-contrast image in axial plane perpendicular to the aqueduct of Sylvius (circle). **a** Rephrased-image. **b**, **c** Phase image through systole and diastole. **d** Magnitude image. **e** Graph demonstrating CSF flow velocity wave

**Fig. 6:**
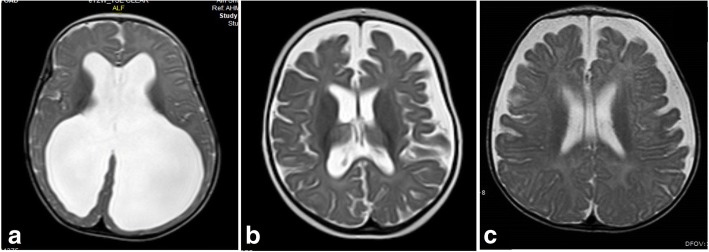
Axial T2-WI in different patients with ventriculomegaly **a** MRI of a 7-month-old girl with hydrocephalus associated with septo-optic dysplasia showing ventricular dilatation with effacement of extra-axial CSF spaces. **b** MRI of an 8-month-old boy with delayed development and microcephaly showing ventricular dilatation ex-vacuo; both lateral ventricles and extra-axial CSF spaces are dilated. **c** MRI of a 5-month-old girl with macrocephaly with normal development showing benign enlargement of subarachnoid spaces


Cine phase-contrast sequence can demonstrate CSF pulsatile flow throughout the cardiac cycle. It can be acquired in sagittal section to monitor the CSF flow through the aqueduct and basal subarachnoid spaces (qualitative assessment). Also, it can be acquired in axial section for quantification of CSF flow (quantitative assessment) (Figs. 4, 5, and 6; Additional file 1: Video S1 and Additional file 2: Video S2) [3].

**Additional file 1: Video S1.** Cine phase-contrast sequence (magnitude images) demonstrates CSF flow as a bright signal throughout the cardiac cycle. (MP4 418 kb)



**Additional file 2: Video S2.** Cine phase-contrast sequence (phase images) demonstrates CSF flow changes from positive to negative and back to positive throughout the diastolic and systolic phases of the cardiac cycle. (MP4 392 kb)


In the phase-contrast technique, two data sets with opposite sensitization are acquired. When the two data sets are subtracted, the signal of the stationary protons is eliminated and flowing nuclei accumulate a net phase proportional to their velocity. The anticipated maximum velocity must be entered into the pulse sequence protocol before phase-contrast data are acquired [4]. After sequence acquisition, images are retrieved in sets of three different contrasts for each plane: re-phased image (magnitude of flow compensated signal), phase image (phase of difference signal), and magnitude (magnitude of difference signal). Since it reflects the phase shift, the phase image is more sensitive to CSF flow detection than the magnitude image [3, 4, 9]. The acquisitions are synchronized with the cardiac cycle generating images with velocity information providing the ability to calculate stroke volume, mean velocity, and peak flow in systole and diastole. The results can be represented via graphical plot [10–15]. Table 3 shows the normal values of CSF flow indices measured across the aqueduct of Sylvius.

**Table 3:** The normal values of CSF flow indices in the aqueduct of Sylvius measured by phase-contrast MRI

CSF flow indices	Definition	Normal values
Stroke volume	The average of the volume passing distally in systole and proximally in diastole [4, 13]	30 to 50 μl [4, 14]
Net flow	Difference between the integrated CSF inflow and outflow over one cardiac cycle [11]	• 0.41 ± 0.51 ml/m in children less than 2 years of age • 0.78 ± 0.89 ml/m in older children [11]
Peak velocity	Either maximum velocity or minimum velocity, whichever has the highest absolute value [15]	• 5.28 ± 2.88 cm/s in infants • 6.57 ± 3.41 cm/s in children • 4.12 ± 2.07; 7.89 ± 2.57 cm/s in adolescents [10, 12]
Mean velocity	The mean CSF flow velocity [15]	• 0.74 ± 0.90 cm/s in infants • 0.87 ± 0.69 cm/s in children • 0.85 ± 0.62 cm/s in adolescents [10]

## MRI findings in CSF flow disorders

### General considerations

One of the ongoing challenges in neuroradiology is to differentiate hydrocephalus from ventricular enlargement associated with brain atrophy (ventriculomegaly ex-vacuo) especially in children, as the sizes of the ventricles and subarachnoid spaces have variable size over the first 2 years of life. Imaging findings should be interpreted in the context of clinical data in order to reach a firm diagnosis of active hydrocephalus. Knowledge of the infant’s head size is essential; a large head or too rapidly enlarging head suggests hydrocephalus, whereas a small or diminishing head circumference is more compatible with atrophy [6].

**Fig. 7:**
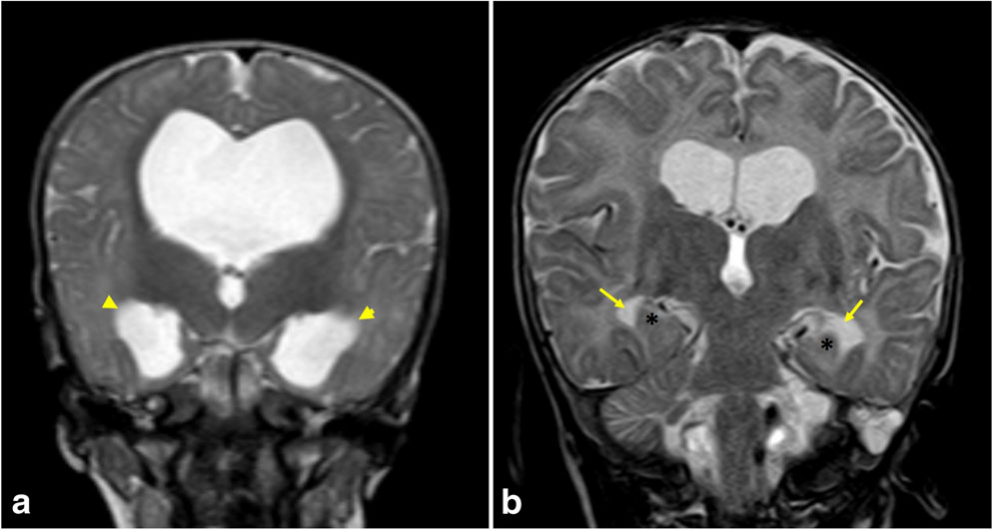
The value of coronal T2-WI in two different patients. **a** MRI of a 7-month-old girl with hydrocephalus showing commensurate dilatation of the temporal horns (arrowheads) with the lateral ventricles. **b** MRI of a 5-month-old boy with brain atrophy. The temporal horns (arrows) are small compared to the bodies of the lateral ventricles; they retain their normal shape and the hippocampi are not displaced medially (asterisk)

**Fig. 8:**
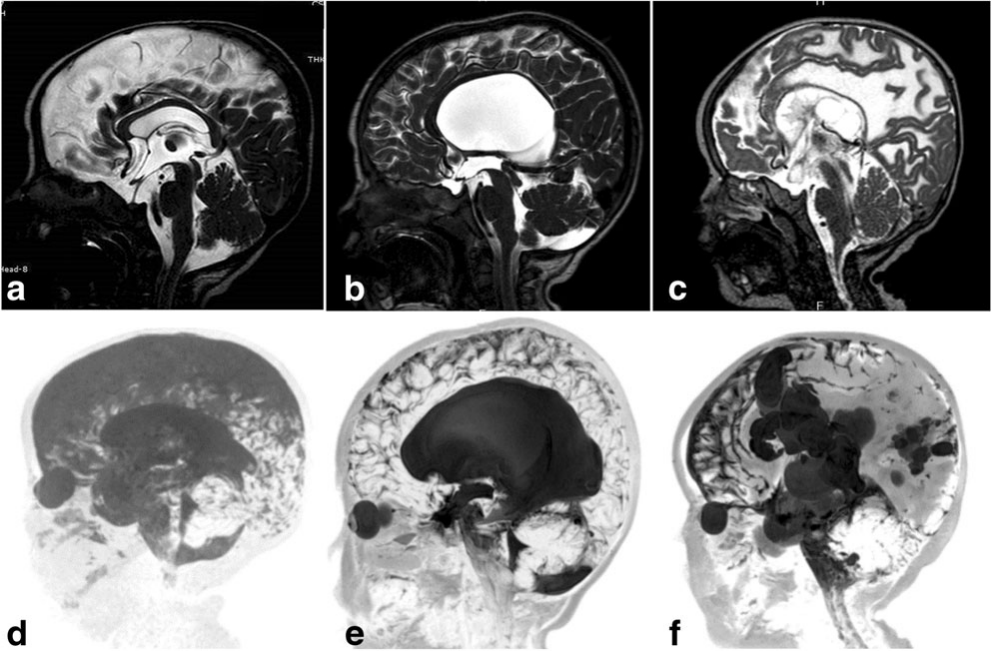
Sagittal 3D-DRIVE (**a**, **b**, and **c**) and maximum intensity projection (MIP) images (**d**, **e**, and **f**) in three different patients. **a**, **d** A 5-month-old girl with cerebral atrophy shows concave lamina terminalis and tuber cinereum with dilated cortical sulci in the MIP image. **b**, **e** A 4-year-old boy with hydrocephalus secondary to aqueduct stenosis demonstrates dilated third and lateral ventricles (note the convex lamina terminalis and tuber cinereum and narrowed cortical sulci in MIP image). **c**, **f** A 22-month-old boy with multiple adhesions in cystic spaces representing entrapped CSF cysts as a result of inflammatory adhesions. Note the narrowed cortical sulci and multiple loculations in the MIP image

Coronal T2-WI and high-resolution T2-WI (3D-DRIVE) can help differentiate hydrocephalus and ventriculomegaly ex-vacuo. In cases of hydrocephalus, coronal T2-WI demonstrates commensurate dilatation of the temporal horns with the lateral ventricles associated with rounding of the lateral angles and medial displacement of the hippocampus. Effacement of pericerebral CSF spaces above the level of obstruction is also demonstrated. However, in cases with ventriculomegaly ex-vacuo, the temporal horns (although large) are small compared to the bodies of the lateral ventricles; they retain their normal shape and the hippocampi are not displaced medially (Fig. 7). In addition, the pericerebral spaces remain visible (Fig. 8). However, in pediatric patients, assessment of pericerebral CSF spaces can be misleading as both atrophy and hydrocephalus can cause enlargement of the ventricles and sulci without effacement [5, 6, 16]. On the other hand, high-resolution T2-WI (3D-DRIVE) demonstrates widening of the third ventricular recesses (chiasmatic, sellar, and pineal recesses) and the downward bulge of the floor of the third ventricle with decrease in the mamillo-pontine distance. These are considered reliable signs for differentiating hydrocephalus from other causes of ventriculomegaly (Fig. 8) [5, 6, 8].

### Specific categories of CSF flow disorders

#### Obstructive hydrocephalus

A consensus classification of hydrocephalus has been proposed to relate most cases of hydrocephalus to obstruction of the CSF pathway, reflecting the surgical approach to hydrocephalus that aims to divert the CSF between the site of production and absorption [17, 18].

**Fig. 9:**
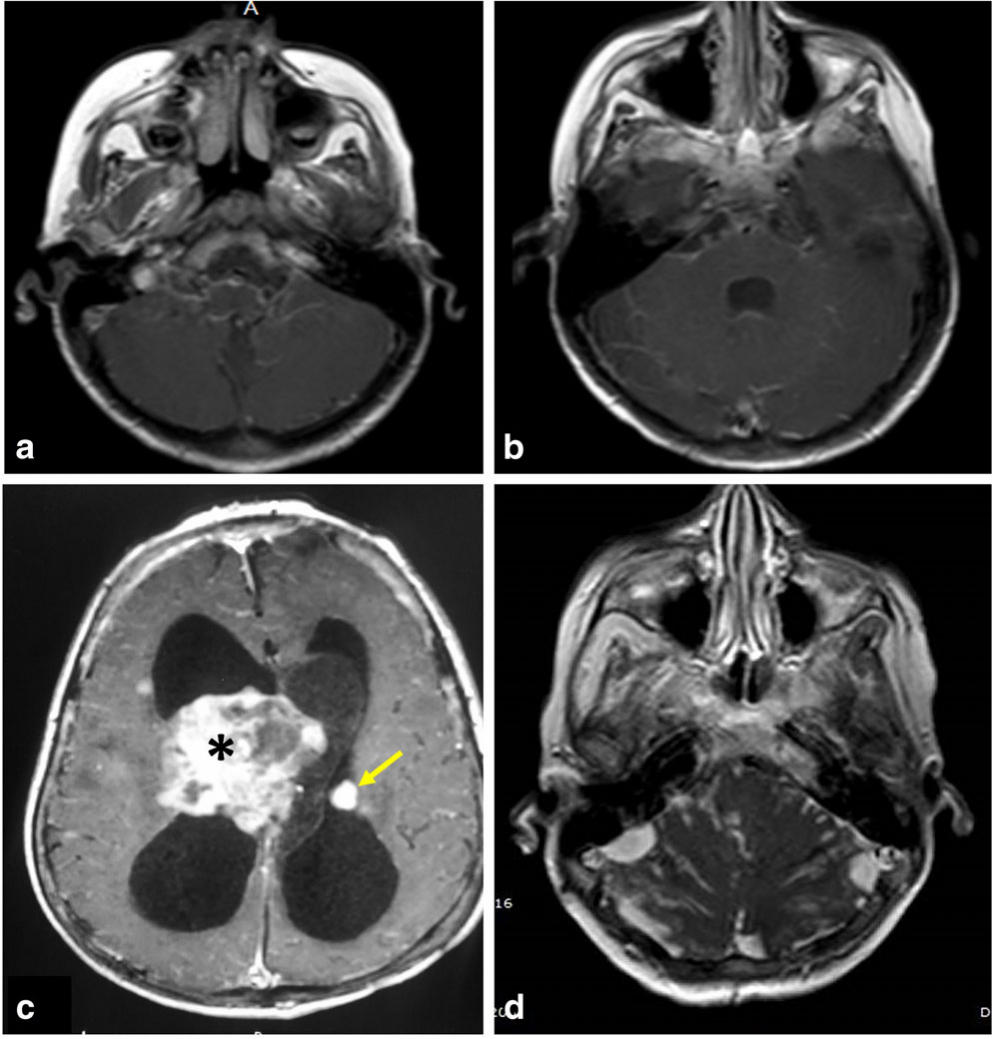
The value of post-contrast T1-WI in different cases with hydrocephalus. **a**, **b** MRI of a 16-year-old boy with hydrocephalus secondary to meningitis demonstrating smooth meningeal enhancement. **c** MRI of an 8-year-old girl with tuberous sclerosis demonstrating enhanced giant cell astrocystoma (asterisk) and subependymal nodule (arrow). **d** MRI of a 5-year-old girl with medulloblastoma showing nodular enhancement representing meningeal metastasis

Obstructive hydrocephalus can be related to purulent or tuberculous meningitis. In the acute stage, MR assessment targets the evidence of infection or its complication via demonstration of leptomeningeal enhancement after contrast administration (Fig. 9). At the late stage, an MRI study demonstrates the site of obstruction and CSF circulation abnormalities, as well as infection-related parenchymal changes [19, 20].

Midline and midline-compressing brain tumors can obstruct the CSF pathway as well. A contrast-enhanced study is indicated in these instances (Fig. 9). Among brain tumors, posterior fossa tumors (e.g., medulloblastoma and ependymoma) are more common in children. Neuroblastoma and medulloblastoma are among the pediatric malignancies that produce leptomeningeal carcinomatosis impairing CSF flow and absorption [20].

Intraventricular hemorrhage in premature infants is the leading cause of pediatric hydrocephalus. T2* demonstrates blood and blood residue. Blood in the acute phase and hemosiderin and scarring in the chronic phase obstruct the ventricles, basilar cisterns, and arachnoid granulation, impairing CSF flow and absorption [20, 21].

**Fig. 10:**
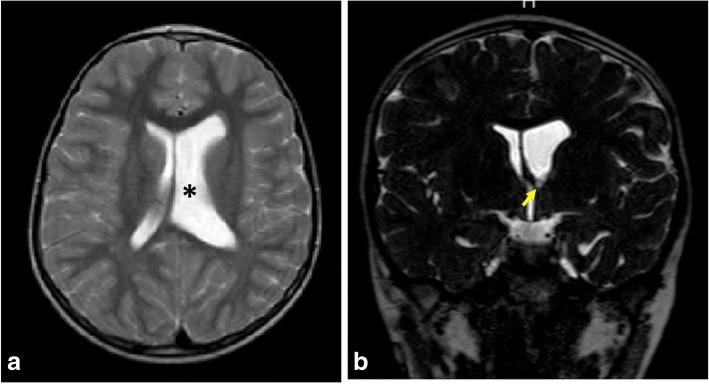
MRI of a 6-year-old boy with foramen of Monro stenosis. **a** Axial T2-WI showing dilated left lateral ventricle (asterisk). **b** Coronal 3D-DRIVE demonstrating foramen of Monro stenosis (arrow)

**Fig. 11:**
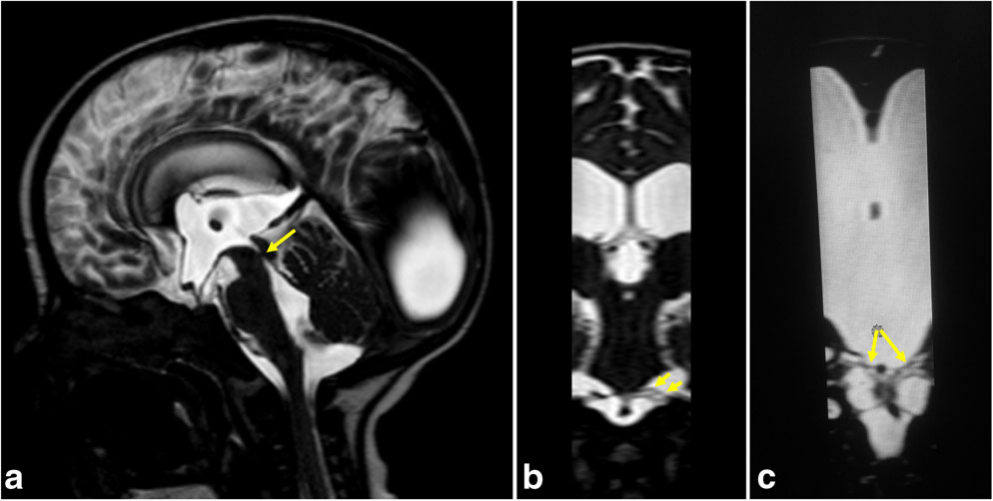
A 7-month-old girl with hydrocephalus secondary to focal aqueduct stenosis. **a** Sagittal 3D-DRIVE demonstrates aqueduct stenosis (arrow) with dilated ventricular system. **b**, **c** Coronal reformatted image reveals prepontine adhesions (arrows)

Obstruction can be encountered in the aqueduct of Sylvius, fourth ventricular exit foramina, and foramen of Monro (Fig. 10) or secondary to synechiae at the ventricles or basal cisterns (Fig. 11). The aqueduct of Sylvius is the most prevalent site of obstruction [5, 16, 18]. This can be related to its narrow caliber. This can occur secondary to congenital stenosis, web or post-inflammatory adhesions, and external compression (e.g., tectal glioma).

**Fig. 12:**
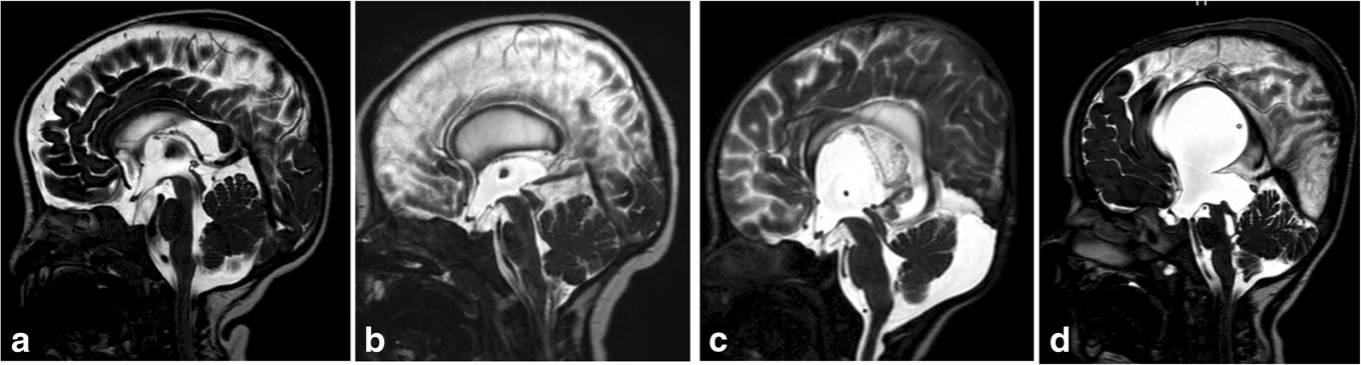
Midsagittal 3D-DRIVE showing the aqueduct of Sylvius and third ventricular anatomy in a normal infant (**a**) and patients with aqueduct stenosis (**b**–**d**). **a** A normal third ventricle of a 1-year-old girl. The lamina terminalis and tuber cinereum are concave centrally which may suggest that the mean pressure in the third ventricle may be lower than that in the cisterns. Note the signal void within the patent aqueduct of Sylvius. **b** MRI of a 3-month-old with mild hydrocephalus showing dilatation of the third ventricle recesses with bulge of the lamina terminalis secondary to multifocal aqueduct stenosis. **c** An 8-month-old girl with hydrocephalus secondary to congenital aqueduct stenosis (note the proximal funneling) showing more dilatation of the third ventricle recesses and more bulge of the lamina terminalis and third ventricle floor. **d** A 5-year-old boy with severe hydrocephalus with effacement of the chiasmatic and suprasellar recesses of the third ventricle. Note dilatation of the pineal recesses

**Fig. 13:**
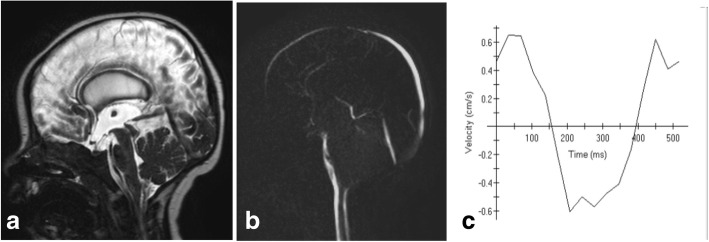
A 3-month-old boy with hydrocephalus. **a** Sagittal 3D-DRIVE demonstrates aqueduct stenosis. **b** Phase-contrast MRI shows absence of detectable flow at the region of the aqueduct with markedly diminished velocity (**c**)

The criteria suggesting aqueduct stenosis include a small fourth ventricle with dilated third and lateral ventricles out of proportion to cortical atrophy. However, these have been described as non-reproducible parameters [20]. 3D-DRIVE can demonstrate the obstructed/stenosed aqueduct (Fig. 12) and precisely describe its shape (either tubular narrowing, focal obstruction/stenosis, associating proximal funneling) (Fig. 12) [5, 16]. In addition, phase-contrast imaging supports the diagnosis of CSF flow obstruction at the level of the aqueduct which aids in the diagnosis of aqueduct stenosis if obstructive hydrocephalus is clinically or radiologically suspected (Fig. 13). The mean value of peak CSF velocity at the aqueduct of Sylvius was found to be diminished (2.38 cm/s) in cases of aqueductal stenosis and near average (5.16 cm/s) in cases with obstruction above the aqueduct. Accelerated flow (12.8 cm/s) was found in cases with infra-aqueductal obstruction [5]. Care should be taken when interpreting velocity measurements, as accelerated flow may be encountered in cases with incomplete aqueductal obstruction, and phase-contrast images should be interpreted in conjunction with high-resolution 3D sequences (3D-DRIVE) [5].

#### Communicating hydrocephalus

**Fig. 14:**
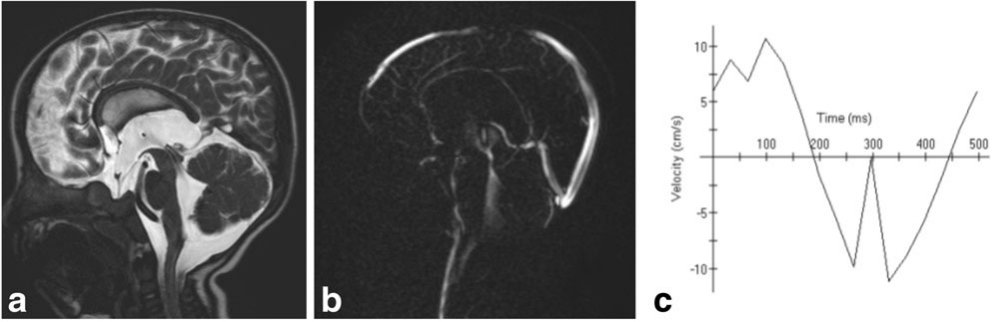
MRI of a 16-month-old boy with communicating hydrocephalus reveals hyperdynamic CSF flow through the aqueduct of Sylvius. **a** Sagittal 3D-DRIVE demonstrates flow void through the aqueduct of Sylvius. **b** Phase-contrast MRI of another patient demonstrates prominent CSF flow through the aqueduct of Sylvius with accelerated CSF flow velocity (**c**)

Communicating hydrocephalus implies cases with no obstruction in the ventricles or the cisterns. Aqueductal CSF flow void is increased in communicating hydrocephalus plausibly because of decreased intracranial compliance (Fig. 14) [20]. Aqueductal flow was found to be 0.56 ± 0.55 ml/min in children with communicating hydrocephalus below the age of 2 years and 5.56 ± 4.73 in older children [11].

In children, the apparently non-obstructive hydrocephalus has been attributed to an imbalance of CSF secretion and absorption. Two patterns have been identified: the benign idiopathic external hydrocephalus (which is more common) and the choroid plexus papilloma-associated hydrocephalus (which is much rarer) [20].

**Fig. 15:**
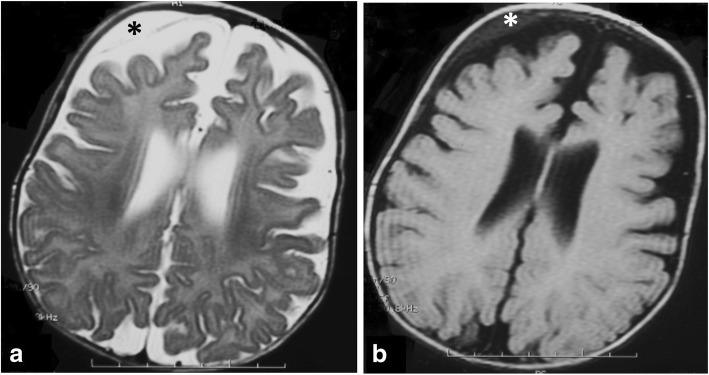
Axial T2-WI (**a**) and FLAIR (**b**) of a 5-month-old girl with glutaric aciduria and macrocephaly showing ventriculomegaly with subdural collection (asterisk)

Benign idiopathic external hydrocephalus is also known as benign enlargement of subarachnoid spaces in infancy. It is characterized by macrocephaly with dilatation of the anterior subarachnoid spaces and ventricular system to a lesser extent. The extra-axial CSF spaces should have CSF signal in all pulse sequences and be traversed by the bridging veins (Fig. 6). These features should be fulfilled in order to be differentiated from prominent extra-axial spaces secondary to subdural hemorrhage especially in patients who have macrocephaly associated with glutaric aciduria type 1 (Fig. 15) [22].

Choroid plexus papilloma-related hydrocephalus is assumed to be related to high secretion of CSF that overwhelms the capacity of absorption. The tumor predominantly affects infants and young children. The tumor usually appears as an avidly enhancing mass floating within the ventricular atrium [20].

#### CSF disorders associated with posterior fossa malformations

##### Dandy-Walker spectrum of malformations

Dandy-Walker complex represents a continuum spectrum of posterior fossa malformations that are characterized by a posterior fossa cyst associated with variable degrees of abnormal development of the cerebellar vermis. The classic Dandy-Walker malformation, Blake’s pouch cyst, posterior fossa arachnoid cyst, mega cisterna, and vermian hypoplasia represent steps on this continuum. Phase-contrast sequence has value in studying CSF flow between CSF spaces in the posterior fossa and the posterior cervical subarachnoid space [23].

**Fig. 16:**
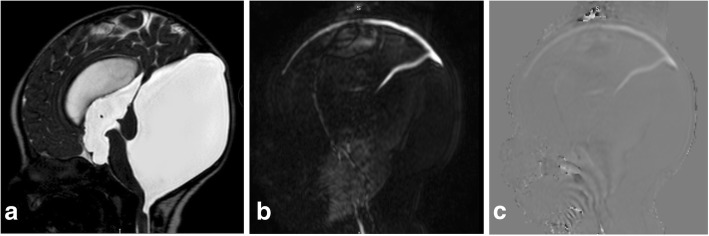
A 3-month-old boy with Dandy-Walker malformation with hydrocephalus. **a** 3D-DRIVE showing large posterior fossa, cerebellar hypoplasia, and stenosis of the aqueduct of Sylvius. **b**, **c** Phase-contrast sequence showing absence of flow through the aqueduct of Sylvius with no communication between the posterior fossa cyst and the posterior cervical subarachnoid space

The typical Dandy-Walker malformation constitutes a large posterior fossa cyst communicating with the fourth ventricle in association with partial or complete agenesis of the cerebellar vermis. A phase-contrast study demonstrates no flow between the posterior fossa cystic space and the posterior cervical subarachnoid space (Fig. 16). Hydrocephalus is associated with typical Dandy-Walker malformation in about 90% of patients. In such conditions, the posterior fossa cyst can be shunted directly. Moreover, aqueduct stenosis is frequently encountered in association with Dandy-Walker malformation (Fig. 16) and a separate ventriculoperitoneal shunt should be placed [23].

**Fig. 17:**
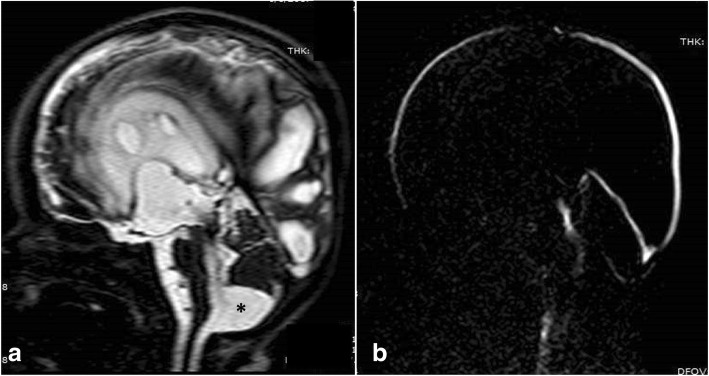
A 12-month-old girl with Blake’s pouch cyst. **a** 3D-DRIVE showing infracerebellar cyst (asterisk) associated with dilated third and fourth ventricles. **b** Phase-contrast MRI reveals absence of communication between the cyst and the posterior cervical subarachnoid space

Blake’s pouch cyst appears as an infracerebellar or retrocerebellar arachnoid cyst associated with tetraventricular hydrocephalus. A phase-contrast study reveals lack of communication between the cyst and the posterior cervical subarachnoid space (Fig. 17) [3, 5, 24].

A posterior fossa arachnoid cyst appears as a well-defined extra-axial cyst or fluid collection that is associated with a normal vermis. It may produce mass effect on the cerebellum or vermis with remodeling of the overlying skull bone. A phase-contrast study can demonstrate whether the cyst is communicating to the CSF spaces which is important in preoperative planning [3, 24].

Mega cisterna magna represents focal enlargement of the subarachnoid space in the posterior and inferior portions of the subarachnoid space with a normal vermis, cerebellum, and fourth ventricle. Phase contrast reveals free communication with the fourth ventricle and posterior cervical subarachnoid space [3, 24].

##### Anomalies of the craniocervical junction (Chiari malformation)

**Fig. 18:**
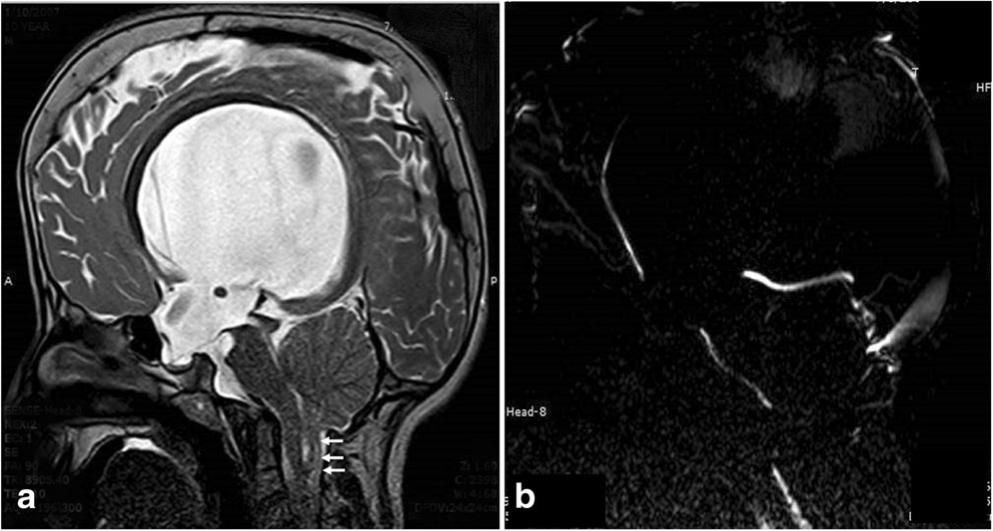
A 10-year-old boy with Chiari malformation. **a** Sagittal T2-WI showing cerebellar tonsil herniation. **b** Phase-contrast MRI demonstrates obstructed CSF flow at the foramen magnum

They represent hindbrain anomalies with caudal displacement of the cerebellar tonsil through the foramen magnum causing plugging of the CSF pathway (Chiari I malformation) and can be associated with myelomeningocele (Chiari II malformation). A phase-contrast study provides information about the extent of CSF flow blockage at the craniocervical junction (Fig. 18) [3, 4, 20]. Quantitative analysis revealed that peak systolic velocity was significantly elevated at the foramen magnum in patients with Chiari I malformation; local flow jets and regions of bidirectional flow were also described [25–27].

#### Assessment of treated hydrocephalus

##### Follow-up after third ventriculostomy

**Fig. 19:**
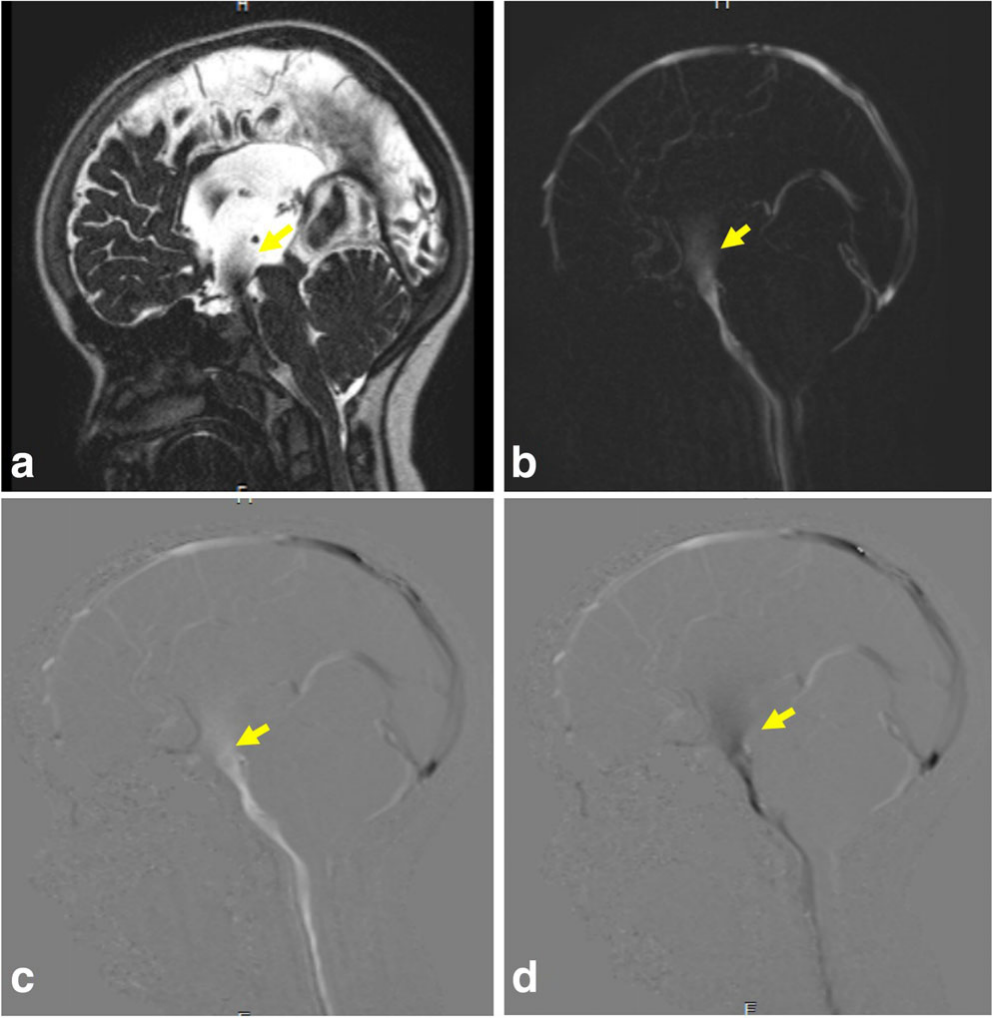
A 9-year-old girl with ventriculostomy. **a** 3D-DRIVE demonstrates flow void through a defect in the third ventricular floor (arrow). **b** Phase-contrast MRI reveals flow jet through the ventriculostomy (arrow). **c**, **d** Phase images through systole and diastole demonstrate the pulsatile flow throughout the cardiac cycle (arrow)


Phase-contrast and 3D-DRIVE sequences have high confidence in the assessment of the flow through the floor of the third ventricle with proper identification of ventriculostomy malfunction (Fig. 19, Additional file 3: Video S3) [28]. Along with the presence of flow void across the floor of the third ventricle, quantitative analysis by measuring overflow amplitude (systolic stroke volume plus net diastolic stroke volume) was found to be a reliable indicator for functional assessment of third ventriculostomy. A high value of more than 75 mm^3^ indicates effective operation with patient improvement. A decrease of stroke volume during follow-up could indicate ventriculostomy malfunction and clinical deterioration [29].

**Additional file 3: Video S3.** Cine phase-contrast sequence in a patient who underwent ventriculostomy demonstrates the jet of flow through the floor of the third ventricle denoting functioning ventriculostomy. (MP4 425 kb)


##### Follow-up after ventriculoperitoneal shunt

**Fig. 20:**
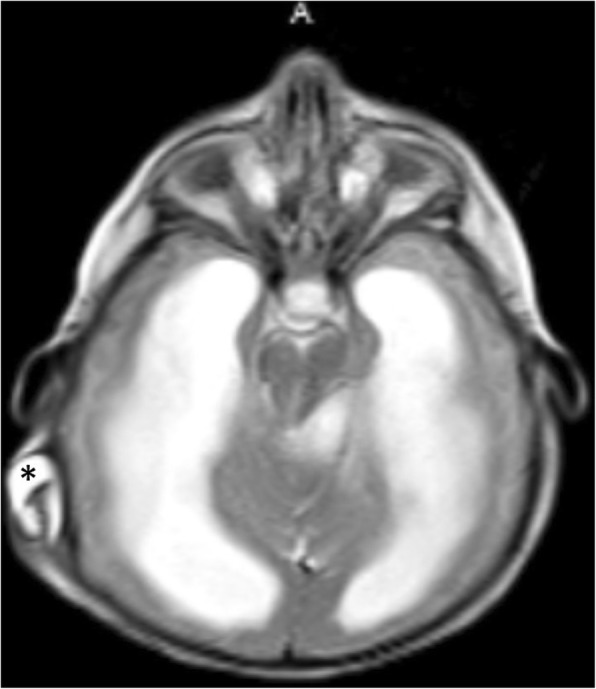
Axial T2-WI of a 6-month-old girl with hydrocephalus and non-functioning ventriculoperitoneal shunt demonstrates fluid surrounding the VP shunt (asterisk)

The diagnosis of shunt malfunction depends mainly on CT of the cranium along with clinical symptoms (headache, vomiting, altered sensorium, feeding difficulty, and fever) [30]. However, clinical symptoms are often vague with equivocal radiological findings, in addition to the burden of frequent radiation exposure. Ventricular enlargement compared to prior examinations is the primary sign for shunt malfunction. However, shunt failure can occur without definite ventriculomegaly. Secondary signs of acute shunt malfunction include transependymal permeation of CSF and pericatheter edema. Subgaleal fluid collections can be encountered (Fig. 20) [31]. Rapid brain MRI protocols have been utilized based on single-shot T2-WI [32]. Recently, it was documented that phase-contrast sequence can be useful in the assessment of functional status of the VP shunt through velocity measurements across the shunt tube [33].

## Conclusion

MRI with the advent of high-resolution T2-WI (3D-DRIVE) and phase-contrast sequences is a crucial imaging modality for the assessment of CSF motion alterations in various disorders which is important for proper patient management.
